# Identification and characterization of Miscanthus yellow fleck virus, a new polerovirus infecting *Miscanthus sinensis*

**DOI:** 10.1371/journal.pone.0239199

**Published:** 2020-09-17

**Authors:** Stephen Bolus, Martha Malapi-Wight, Samuel C. Grinstead, Irazema Fuentes-Bueno, Leticia Hendrickson, Rosemarie W. Hammond, Dimitre Mollov

**Affiliations:** 1 USDA-ARS, National Germplasm Resources Laboratory, Beltsville, Maryland, United States of America; 2 USDA-APHIS-PPQ, Plant Germplasm Quarantine Program, Beltsville, Maryland, United States of America; 3 USDA-APHIS-BRS, Biotechnology Risk Analysis Programs, Riverdale, Maryland, United States of America; 4 USDA-ARS, Molecular Plant Pathology Laboratory, Beltsville, Maryland, United States of America; Louisiana State University, UNITED STATES

## Abstract

*Miscanthus sinensis* is a grass used for sugarcane breeding and bioenergy production. Using high throughput sequencing technologies, we identified a new viral genome in infected *M*. *sinensis* leaf tissue displaying yellow fleck symptoms. This virus is most related to members of the genus *Polerovirus* in the family *Luteoviridae*. The canonical ORFs were computationally identified, the P3 coat protein was expressed, and virus-like particles were purified and found to conform to icosahedral shapes, characteristic of the family *Luteoviridae*. We propose the name Miscanthus yellow fleck virus for this new virus.

## Introduction

*Miscanthus sinensis* Andersson (Chinese silvergrass) is an herbaceous perennial grass native to Eastern Asia. It is naturalized in parts of the United States [1] and is currently being investigated for bioenergy [2] and sugarcane breeding purposes [3]. *Miscanthus sinensis* is susceptible to several diseases caused by viruses, including: sorghum mosaic virus [4], switchgrass mosaic virus [5], and barley yellow dwarf virus [6, 7] and can also serve as a host to the aphid species *Rhopalosiphum maidis* [6]. Sugarcane (*Saccharum* spp.) is an important food source and a bioenergy crop, and its cultivation is restricted to subtropical and tropical climates. Currently, there are efforts to expand its cultivation to cooler climates by breeding sugarcane germplasm with the cold-hardy *Miscanthus* spp. [3]. Miscanthus has also been reported to be resistant to several sugarcane pathogens. Hence, these *Miscanthus* x *Saccharum* hybrids, also called “miscanes”, could be used as a source of resistance to the economically important viral pathogen sugarcane yellow leaf virus (ScYLV) [8, 9] and the fungal orange rust pathogen *Puccinia kuehnii* [9], among others.

ScYLV is a causal agent of yellow leaf syndrome in sugarcane, which is characterized by yellowing of the leaf midrib on the abaxial surface, and accounts for yield losses of 11–50% [10]. ScYLV is spread in a persistent, circulative, and non-propagative manner by several aphid species, notably by *Melanaphis sacchari* and *R*. *maidis* [11, 12]. ScYLV is also spread by infected stem cuttings or sections of stalks (“setts”) used to propagate sugarcane and to distribute germplasm [10].

ScYLV is a polerovirus member of the *Luteoviridae* [10], a family of plant-infecting, positive-sense RNA viruses that are principally phloem-limited, vectored by aphids, and form icosahedral (t = 3) virus-like particles (VLPs) [13, 14]. Many viruses in the family *Luteovirida*e, besides ScYLV, negatively affect crop production, notably *Barley yellow dwarf virus*, *Cereal yellow dwarf virus*, *Beet western yellows virus*, and *Potato leafroll virus* [13]. The family is currently divided into three genera: *Luteovirus*, *Polerovirus*, and *Enamovirus*, which are broadly separated based on their genome organization [13, 14].

In this work, we identified and characterized a new virus associated with yellow flecks on *Miscanthus sinensis* leaf tissue. The complete genomic sequence was obtained by high throughput sequencing (HTS), 5’ and 3’ Rapid Amplification of cDNA Ends (RACE), and subsequent Sanger sequencing. The coat protein (CP) was cloned and expressed in a potato virus X (PVX) vector, and VLPs were identified in *Nicotiana benthamiana*. This virus is placed in the family *Luteoviridae* and genus *Polerovirus* based on phylogenetic grouping and genomic characteristics. Based on current demarcation criteria, this virus is considered a new species putatively named Miscanthus yellow fleck virus (MYFV).

## Materials and methods

### Plant material, RNA extraction, PCR testing, and high throughput sequencing

A *Miscanthus sinensis* accession was imported from South Korea in 2015 to the United States Department of Agriculture (USDA) Animal and Plant Health Inspection Service (APHIS) Plant Germplasm Quarantine Program (PGQP) in Beltsville, Maryland. As part of the routine indexing performed by PGQP, the imported grass accession was established in quarantine greenhouse settings and observed for symptom development. The plants from the same accession exhibited foliar yellow flecks and were tested by reverse-transcription (RT) PCR for multiple regulated RNA viruses. The viral pathogens that the *M*. *sinensis* was tested for included group tests for luteoviruses (including two ScYLV specific tests), potexviruses, carlaviruses, closteroviruses, potyviruses, and mastreviruses. Total RNA was extracted from pooled leaf tissue representing several plants form the same accession using the Qiagen RNeasy Plant Mini Kit (Qiagen; MD, USA) following manufacturer’s instructions. For the detection of ScYLV, RT-PCR tests included the identification of the CP using primers YLS 462 and YLS 111 [15] and the Luteovirus Group PCR test from Agdia (Agdia; IN, USA).

In January 2017, at the USDA Agriculture Research Service (ARS) National Germplasm Resources Laboratory (NGRL), total RNA was extracted from symptomatic leaves collected from a few plants derived from the same accession previously tested by PGQP using the Qiagen RNeasy Plant Mini Kit per manufacturer’s instructions. The RNA was outsourced to SeqMatic (CA, USA), where it was subjected to DNase treatment, rRNA depletion, and cDNA library construction. The library was sequenced on an Illumina NextSeq 500 platform as single end, 75-base pair (bp) reads (1 x 75). The reads were trimmed and *de novo* assembled into contigs using CLC Workbench 10.1.1 (CLC Bio; Qiagen; USA) with parameters of 200 nucleotide (nt) minimum contig length and automatic bubble and word size.

In April 2017 at the PGQP, total RNA was re-extracted from pooled, symptomatic leaves using a Qiagen RNeasy Plant Mini Kit with RNase-free DNase I Set (Qiagen; MD, USA). The integrity of the DNase-treated RNA was assessed with the 4200 TapeStation RNA ScreenTape Assay (Agilent Technologies; CA, USA), and the concentration was fluorometrically-quantified with the Qubit 3.0 fluorometer RNA BR Assay (Thermo Fisher Scientific; MA, USA). The single-indexed cDNA library was constructed as previously reported [16], validated, and quantified using the 4200 TapeStation D1000 HS dsDNA Assay and Qubit fluorometer dsDNA HS Assay, respectively. High throughput sequencing of the cDNA library was performed in-house on an Illumina NextSeq 500 platform to generate single end, 75-bp reads (1 x 75). Upon removal of adaptors and indices, reads with a quality score ≤ 0.001, i.e., Phred Q30, and a maximum of two ambiguous nucleotides (nts) were retained. The trimmed reads were de novo assembled into contigs with CLC Genomics Workbench 10.1.1 using the same parameters as described above.

### Whole genome assembly and analysis

Terminal sequences at the 5’ end were amplified with a RACE kit (Invitrogen; USA) using genomic specific and AAP primers (Table 1). The 3’ end sequences were amplified by first polyadenylating the 3’ end of the viral genome with poly (A) polymerase (New England Biolabs; MA, USA) followed by cDNA synthesis using SuperScript™ III First-Strand Synthesis System (Invitrogen) with M4T primer (Table 1). Next, 3’ end sequences were further amplified using GoTaq® Green Master Mix (Promega; WI, USA) with a genome specific and M4 primer (Table 1) following thermocycler conditions of 1 cycle of 95°C for 2 minutes; 30 cycles of 96°C for 1 minute, 55°C for 45 seconds, and 72°C for 1 minute; and 1 cycle of 72°C for 5 minutes. The amplified products from both 5’ and 3’ end reactions were extracted from agarose gels using the QIAquick Gel Extraction Kit (Qiagen; MD, USA), and the PCR fragments were cloned into the pGEM®-T Easy Vector System (Promega) per the manufacturer’s protocol. The recombinant plasmids were transformed into JM 109 *Escherichia coli* cells. Nucleotide sequences of at least eight individual clones derived from each 5’ and 3’ RACE reaction were determined in both directions using an automated DNA sequencer (MCLAB). Sequence reads were analyzed and aligned with the reference sequence using Geneious v. 9 (Biomatters; New Zealand) to obtain the full viral genome.

**Table 1:** Primer sequences used for 5’ and 3’ Rapid Amplification of cDNA Ends (RACE) and coat protein cloning.

Primers	Sequence (5’ to 3’)	Use
AAP	GGCCACGCGTCGACTAGTACGGGIIGGGIIGGGIIG	5’ RACE
MYFV_SP1_R	CGATGTCGCTTGGTTGATATTC	5’ RACE
MYFV_SP2_R	GAGCAATGATCCAGCCATTTC	5’ RACE
MYFV_SP3_R	CAGCTCTCAACCACGAAACTA	5’ RACE
M4T	GTTTTCCCAGTCACGACTTTTTTTTTTTTTTT	3’ RACE
M4	GTTTTCCCAGTCACGAC	3’ RACE
MYFV_P1_F	GATGACACAAGAGGAGAGGAATG	3’ RACE
MYFV_P2_F	GTCCCACCACATGACGATAAA	3’ RACE
MYFV_CP_F	CCTGCCATGGATACGGGAGGTAGA	Coat protein cloning
MYFV_CP_R	CCTGGAATTCCTATTTCGGACTCTG	Coat protein cloning

I represents deoxyinosine.

Open reading frames (ORFs) were predicted using the National Center for Biotechnology Information (NCBI) Open Reading Frame Finder (https://www.ncbi.nlm.nih.gov/orffinder/). Protein sequences were compared to known sequences using BLASTP and the non-redundant protein sequences database (NCBI).

### Phylogenetic analysis

A dataset was compiled including the complete genomes of all viruses in the family *Luteoviridae* deposited in the RefSeq database (NCBI) along with the complete viral genome of the new Miscanthus virus. Using MEGA X software [17], the sequences were aligned using ClustalW under default settings. A phylogenetic tree was constructed using the Maximum Likelihood method and Tamura-Nei model under default settings [18]. One thousand bootstrap replications were performed, and a condensed tree was built by collapsing branches with less than 50% bootstrap support.

### Heterologous expression of coat protein

For expression of the Miscanthus virus coat protein (CP) in plants, the CP gene was introduced into the plant expression vector, pGD-PVX-MCS [19], via a pSKAS intermediate vector [20]. The primer pair MYFV_CP_F / R (Table 1) was used to amplify the full-length CP gene from the original RNA extract. The amplicon generated was a CP coding region flanked by *Nco*I and *Eco*RI restriction sites. The product was subsequently cloned into the TA cloning vector pCR4 (Invitrogen), following manufacturer’s instructions, to produce pCR4:CPA1. The resultant pCR4:CPA1 was digested with *Nco*I/*Eco*RI restriction enzymes; the insert was gel purified from a 1% agarose/1X TBE gel using the QIAquick gel extraction kit (Qiagen; MD, USA) and ligated into the pSKAS vector, similarly digested with *Nco*I/*Eco*RI, using T4 DNA ligase (New England Biolabs; MA, USA). The ligation mix was transformed into competent Top10 *Escherichia coli* cells (Thermo Fisher Scientific; MA, USA), yielding pSKAS:CPA1. The pSKAS:CPA1 plasmid was digested with *Apa*I/*Spe*I, and the insert was gel purified and cloned into *Apa*I/*Spe*I-digested pGD-PVX-MCS vector [19] resulting in pGD-PVX-MCS:CPA1. The construct was introduced into competent *Agrobacterium tumefaciens* EHA105 cells [20].

For agroinfiltration of the pGD-PVX-MCS:CPA1 construct, a method previously described [21] was used with modifications. Briefly, *Nicotiana benthamiana* plants were used for all agroinfiltration experiments. Bacterial suspensions of *A*. *tumefaciens* strain EHA105 were derived from fresh (1- to 2-day-old) cultures grown on Petri plates of LB media containing 50 μg mL^-1^ rifampicin and 50 μg mL^-1^ kanamycin. For agroinfiltration, a loop of cells was resuspended in 5 mL of LB broth and grown for 1–2 days at 28°C. The bacterial cells were then pelleted and resuspended in 2 mL of MES buffer (10 mm MgCl_2_, 10 mm MES, pH 5.7). Acetosyringone was added to a final concentration of 200 μm. Bacterial suspensions were then kept at room temperature for 2–3 h. To co-infiltrate agrobacteria containing the pGD-PVX-MCS:CPA1 construct, 450 μL of bacteria containing the construct was mixed with 50 μL of similarly treated agrobacteria containing pGD-p19, which express the tomato bushy stunt virus p19 coding region to minimize host RNA silencing [22], prior to infiltration. Infiltrations of 100–200 μL were conducted by gently appressing a 1-mL disposable syringe to the abaxial surface of fully expanded leaves. Following agroinfiltration, *N*. *benthamiana* plants were maintained in the laboratory under fluorescent lighting for 15 days.

### Virus-like particle purification and transmission electron microscopy

Four grams of symptomatic *N*. *benthamiana* leaves were used for VLP purification. Plant material was ground using a mortar and pestle with liquid nitrogen and silicon carbide until powdered. Sixteen mL of 100 mM sodium potassium phosphate buffer (pH 5.8) was added, and the slurry was filtered through heavyweight 50 stabilizer polyester fabric (Pellon; FL, USA). The liquid phase was then centrifuged at 19,800 g_max_ for 10 minutes. The supernatant was collected, and after addition of 5% (v/v) Triton X-100, the mixture was layered over 30% (w/v) sucrose in 100 mM sodium phosphate buffer (pH 5.8), followed by centrifugation at 109,000 g_max_ for 2 hours. The pellet was resuspended with 100 mM sodium phosphate buffer (pH 5.8). Ten μL of the suspension was applied to copper grids coated with carbon and formvar. Grids were stained with 2% phosphotungstic acid and visualized on a Hitachi 7700 Electron Microscope at the ARS Confocal and Electron Microscopy Unit in Beltsville, MD. ImageJ software (National Institutes of Health; MD, USA) was used to determine the average diameter of VLPs (https://imagej.nih.gov/ij/).

## Results and discussion

### Identification of a single virus in *Miscanthus sinensis* leaf tissue

**Fig 1:**
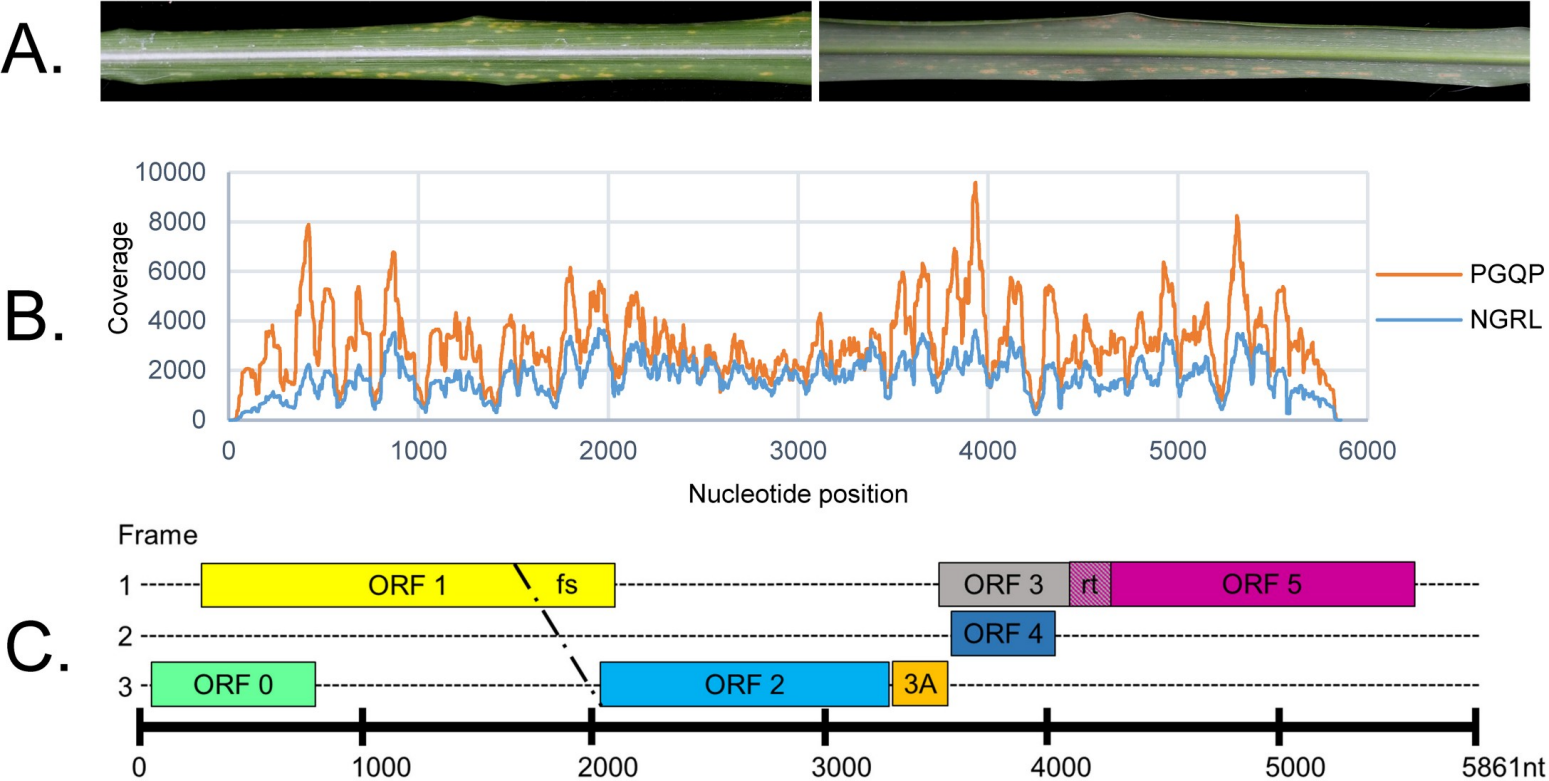
Virus symptoms, sequencing, and genome analysis. (A) Adaxial and abaxial sides of a *Miscanthus sinensis* leaf displaying viral infection symptoms. (B) Depth of coverage at each nucleotide position obtained by high-throughput sequencing. (C) Schematic representation of Miscanthus yellow fleck virus genome organization. Open Reading Frames (ORFs) were identified using ORF Finder (National Center for Biotechnology Information). The nucleotide positions of the ORFs depicted here are as follows in brackets: ORF0 [57–854], ORF1 [250–2100], ORF1-2 [250–3356], ORF3A [3414–3569], ORF3 [3553–4140], ORF4 [3584–4036], and ORF3-5 [3553–5589]. The 5’ untranslated region (UTR), internal UTR, and 3’ UTR are predicted to correspond to nucleotide positions 1–56, 3357–3413, and 5590–5861, respectively. fs denotes the predicted -1 frame shift at nucleotide position 1650, and rt denotes the predicted readthrough stop codon at nucleotide positions 4141–4143. Predicted protein products from ORFs are listed in Table 2. The diagram presented here is approximate and not necessarily drawn to scale.

In 2015, yellow flecks symptoms were observed on the leaves of a *M*. *sinensis* accession that was imported by the USDA-APHIS PGQP program (Fig 1A). As part of the quarantine protocol, the plant accession was indexed for the identification of regulated pathogens using conventional diagnostic techniques (i.e. ELISA, PCR and bioassays). RT-PCR tests identified the grass accession to be infected with ScYLV or a closely related virus of the *Luteoviridae* family (results not shown). However, to date, *M*. *sinensis* has not been reported to be a host for ScYLV [10]. For further diagnostic testing, total RNA was re-extracted and subjected to HTS and bioinformatics analysis. Two independent rounds of HTS performed in separate laboratories confirmed a single virus infection in this introduced accession. A total of 13,196,261 reads were obtained for the extract prepared in the NGRL. Raw data reads were assembled into 34,908 contigs. BLAST analyses revealed one of these contigs as viral, 5840 nt long and with average coverage of 1,722 x per nt position (Fig 1B). Data generated by the PGQP produced 92,221,483 reads that were assembled into 76,032 contigs. BLAST analyses revealed that one of these contigs had similarity to ScYLV with virtually the full genome length and an average coverage at 3,119 x per nt position. No other virus-like contigs were detected in either sequencing run. Both data sets yielded a single viral contig. These contigs were 100% identical when compared at the nucleotide level. Interestingly, despite the different number of reads obtained from each run, there was similar coverage patterns observed between the PGQP and NGRL samples (Fig 1B). The HTS efforts and 5’ and 3’ RACE followed by Sanger sequencing resulted in a high-quality, complete viral genome at 5,861 nts. A BLAST search of the complete genome sequence returned hits to known poleroviruses in the family *Luteoviridae*.

### Genome characterization

To further characterize the genome of the novel polerovirus, herein referred to as Miscanthus yellow fleck virus (MYFV; GenBank accession no. MT520166), open reading frames were computationally identified using Open Reading Frame (ORF) Finder (NCBI) (Fig 1C). The predicted protein products of seven ORFs shared identities with those of other poleroviruses (Table 2) and were further characterized.

**Table 2:** Top match from amino acid sequence comparisons with predicted proteins from Miscanthus yellow fleck virus through blastp searches of the National Center for Biotechnology Information (NCBI) non-redundant protein sequence database.

ORF	Protein	Predicted function	Length (aa)	Top match protein name, organism, accession number (NCBI)	Amino acid identity (%)	Query cover-age (%)	E value
ORF0	P0	RNA silencing suppressor	265	P0, ScYLV, AIN44132.1	47	56	4e-30
ORF1	P1	multi-functional protein	616	P1, ScYLV, ADD84803.1	55	99	0.0
ORF1-2	P1-P2	RNA-dependent RNA polymerase	1035	P1-P2, ScYLV, AYC21700.1	68	99	0.0
ORF3A	P3a	movement protein	51	P3a, ScYLV, AXM90645.1	70	86	1e-13
ORF3	P3	coat protein	196	P3, WLYaV, YP_009407911.1	81	100	5e-84
ORF4	P4	movement protein	150	P4, ScYLV, AEG89080.1	78	100	7e-71
ORF3-5	P3-P5	putative aphid transmission factor	677	P3-P5, WLYaV, YP_009407910.1	60	98	0.0

ScYLV, sugarcane yellow leaf virus; WLYaV, wheat leaf yellowing-associated virus.

ORF0 is predicted to encode for P0, an RNA silencing suppressor [23, 24] that is most similar (47% identity, 56% query coverage, 4e-30 E value) to the P0 from ScYLV (Table 2). The F-box like motif (LPxxL/I), which has been shown to be required for RNA silencing activity [23], was present in P0 of MYFV as VPILC. Some variation was already observed in this motif, including IPIIL for wheat leaf yellowing-associated virus (WLYaV) and VPILL for ScYLV [25].

ORF1 is expected to encode for P1, a multifunctional protein [26] with highest identity (55% identity, 99% query coverage, 0.0 E value) to P1 of ScYLV (Table 2). A putative protease motif H(X_~25_)[D/E](X_70-80_)T[R/K]XGXSG, without conservation of the basic [R/K] that is found among P1s of poleroviruses [26], was also found in the predicted MYFV P1 sequence (results not shown).

**Fig 2:**
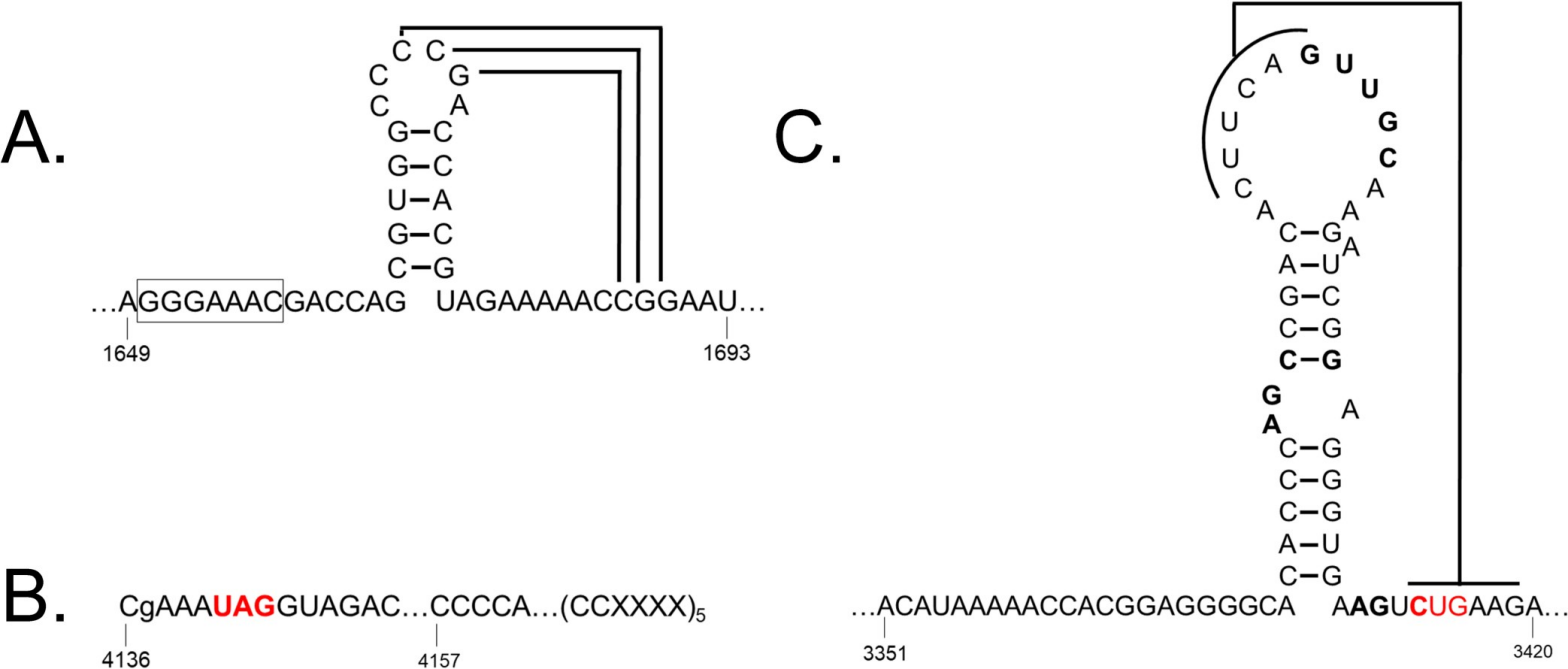
Proposed sites for ribosomal frameshift, ribosomal readthrough, and exoribonuclease-resistant RNA pseudoknot structure for Miscanthus yellow fleck virus. (A) Depiction of potential pseudoknot structure following the “slippery heptanucleotide” sequence (boxed), where ribosomal -1 frameshifting is proposed to occur, connecting open reading frame (ORF) 1 and 2. Drawing was modeled after that presented for sugarcane yellow leaf virus [27]. (B) Sequences flanking the potential readthrough stop codon site (in red), connecting ORFs 3 and 5. Uppercase letters indicate nucleotide residues and repeats flanking the stop codon that were previously shown to be conserved in *Luteoviridae* viruses [26]. X represents any nucleotide. (C) Predicted exoribonuclease-resistant RNA pseudoknot structure for Miscanthus yellow fleck virus that encompasses the internal-UTR. CUG start codon for ORF 3A is highlighted in red. The bolded nucleotides were those found to be conserved in *Tombusviridae* and *Luteoviridae* virus families [31].

ORF1-2 is an expected product combining ORFs 1 and 2 through a predicted -1 ribosomal frameshift at nucleotide position 1650 (Fig 1C). The site of the predicted frameshift was found to contain conserved features previously identified to be important for ribosome frameshifting, including a slippery heptanucleotide sequence and downstream pseudoknot structure (Fig 2A) [26, 27]. ORF1-2 encodes P1-P2, an RNA-dependent RNA polymerase [26], with 68% identity (99% query coverage, 0.0 E value) to P1-P2 from ScYLV (Table 2). The GDD motif [25, 26] is also conserved in the P1-P2 amino acid sequence of MYFV (results not shown).

ORF3A contains a non-canonical start codon CTG and is predicted to encode a polypeptide involved in long distance viral movement [28] with 70% amino acid sequence identity (86% query coverage, 1e-13 E value) to P3a from ScYLV (Table 2). CTG is a non-canonical start site but is the strongest non-ATG one in many systems [28]; in MYFV, it occurs in a favorable context with A at -3 and A at +4, with the most ideal being A at -3 and G at +4 [26, 28].

ORF3 is predicted to encode for the coat protein (P3) [26], which shares 81% identity (100% query coverage, 5e-84 E value) to the P3 sequence of WLYaV (Table 2). P4 is a putative protein product of ORF4, which is predicted to be translated through a leaky scanning mechanism and is most similar (78% identity, 100% query coverage, 7e-71 E value) to P4, a movement protein [26] from ScYLV (Table 2). The stop codon for ORF3 is a potential readthrough stop codon (nucleotide positions 4141–4143), which would result in a large polypeptide product combining ORF3 and 5 (Fig 1C). It contains features known to be important for facilitating readthrough, including conserved nts surrounding the stop codon and the conserved CCCCA plus repeat sequences CCXXXX, where X represents any nucleotide, that are 3’ terminal to the stop codon sequence [26] (Fig 2B). P3-P5, the predicted protein product from ORF3-5 resulting from the readthrough of the ORF3 stop codon, is a putative aphid transmission factor [26] with 60% identity (98% query coverage, 0.0 E value) to P3-P5 from WLYaV (Table 2).

The MYFV genome begins with 5’ ACATAAAA 3’, which is repeated at nucleotide positions 3351–3358. A conserved 5’ ACAAAA 3’ motif is found in many poleroviruses both at the 5’ end and near the beginning of predicted sgRNA sites and is thought to function as a possible transcription enhancer, producing the full genomic RNA and subgenomic RNA species [26, 29, 30]. Although the 5’ ACAAAA 3’ motif is not fully conserved in MYFV, exceptions have already been reported, such as 5’ ACTAAA 3’ for WLYaV [25].

MYFV has 3 predicted untranslated regions (UTRs), including a 56 bp 5’UTR, a 272 bp 3’UTR without a polyA tail, and an internal-UTR of 57 bp from nucleotide positions 3357–3413. The internal-UTR forms a predicted pseudoknot structure that could function as an exoribonuclease-resistant RNA element (Fig 2C). The predicted pseudoknot sequence contains all the base pairs found to be conserved in internal-UTR pseudoknot structures that were shown to be protective against exoribonuclease activity across viruses in the families *Tombusviridae* and *Luteoviridae* [31]. The benefit of having these exoribonuclease-resistant RNA elements near the beginning of an internal-UTR is not fully understood, but they could protect subgenomic RNAs from degradation by 5’-to-3’ cellular exonucleases [31].

**Fig 3:**
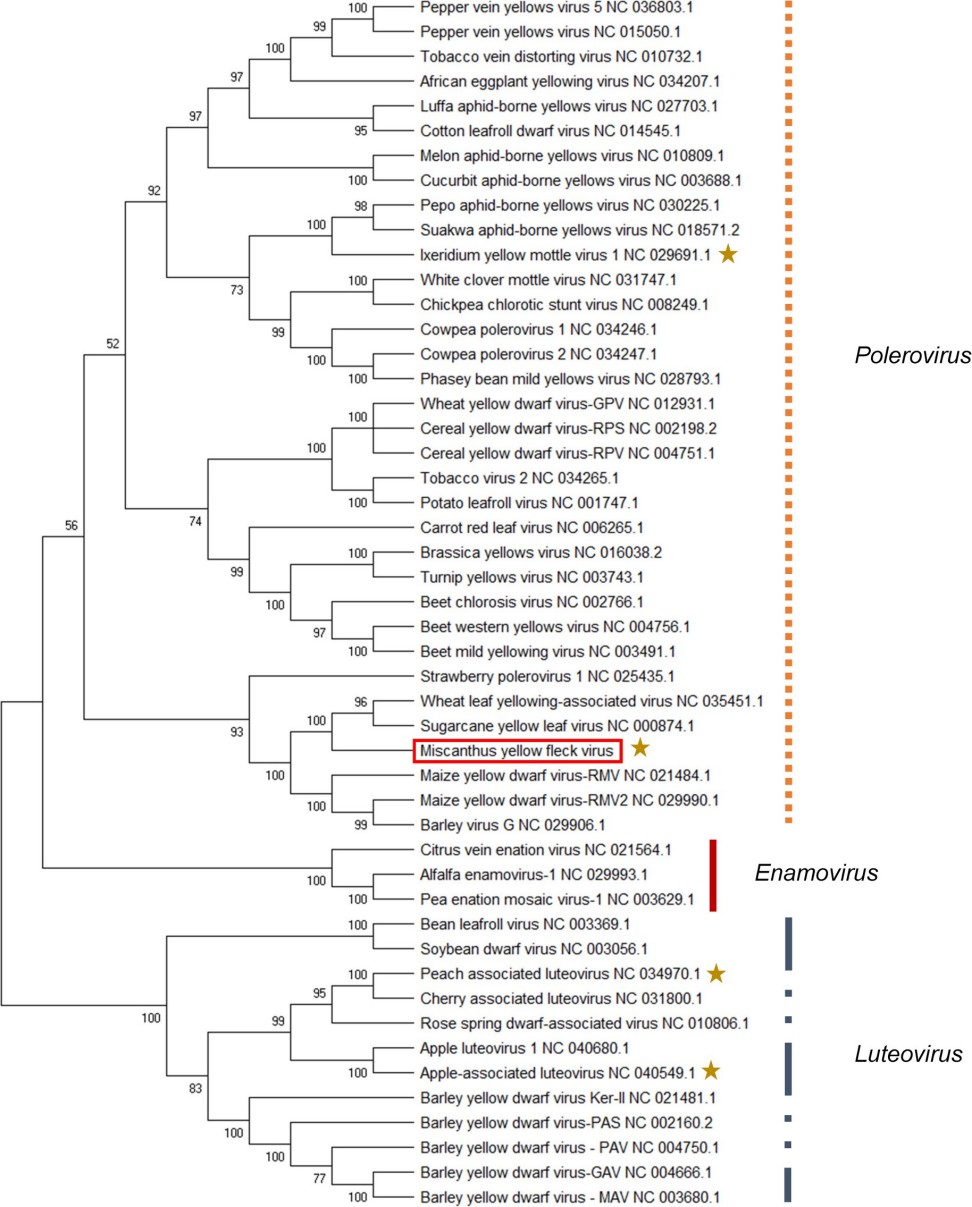
Phylogenetic tree showing the relationship of the complete genome of Miscanthus yellow fleck virus to viruses within the family *Luteoviridae*. Virus names are shown alongside their corresponding NCBI accession numbers. ClustalW was used to align Miscanthus yellow fleck virus with 48 complete viral genome sequences, and there were 7803 positions in the final dataset. The phylogenetic tree was constructed using the Maximum Likelihood method and Tamura-Nei model with MEGA-X software [17]. The numbers next to branches indicate the percentage of 1000 replicate trees in which associated taxa clustered together. Branches with less than 50% bootstrap support were collapsed. *unclassified *Luteoviridae* virus.

To determine the relatedness of MYFV to other viruses in the *Luteoviridae* family, their complete genomes were aligned, and a phylogenetic tree was constructed using Maximum Likelihood (Fig 3). The phylogenetic tree revealed a distinct grouping of luteoviruses that was strongly supported. Enamoviruses grouped together. MYFV was placed within poleroviruses and is sister to a group including ScYLV and WLYaV (Fig 3). At the protein identity level, MYFV was more similar to ScYLV for all predicted protein products with the exception of P3 and P3-5, where it was found to be most similar to WLYaV (Table 2). However, the protein identity difference between WLYaV and ScYLV was within a couple of percentage points for these two protein products (results not presented).

### Functional characterization of the coat protein sequence

**Fig 4:**
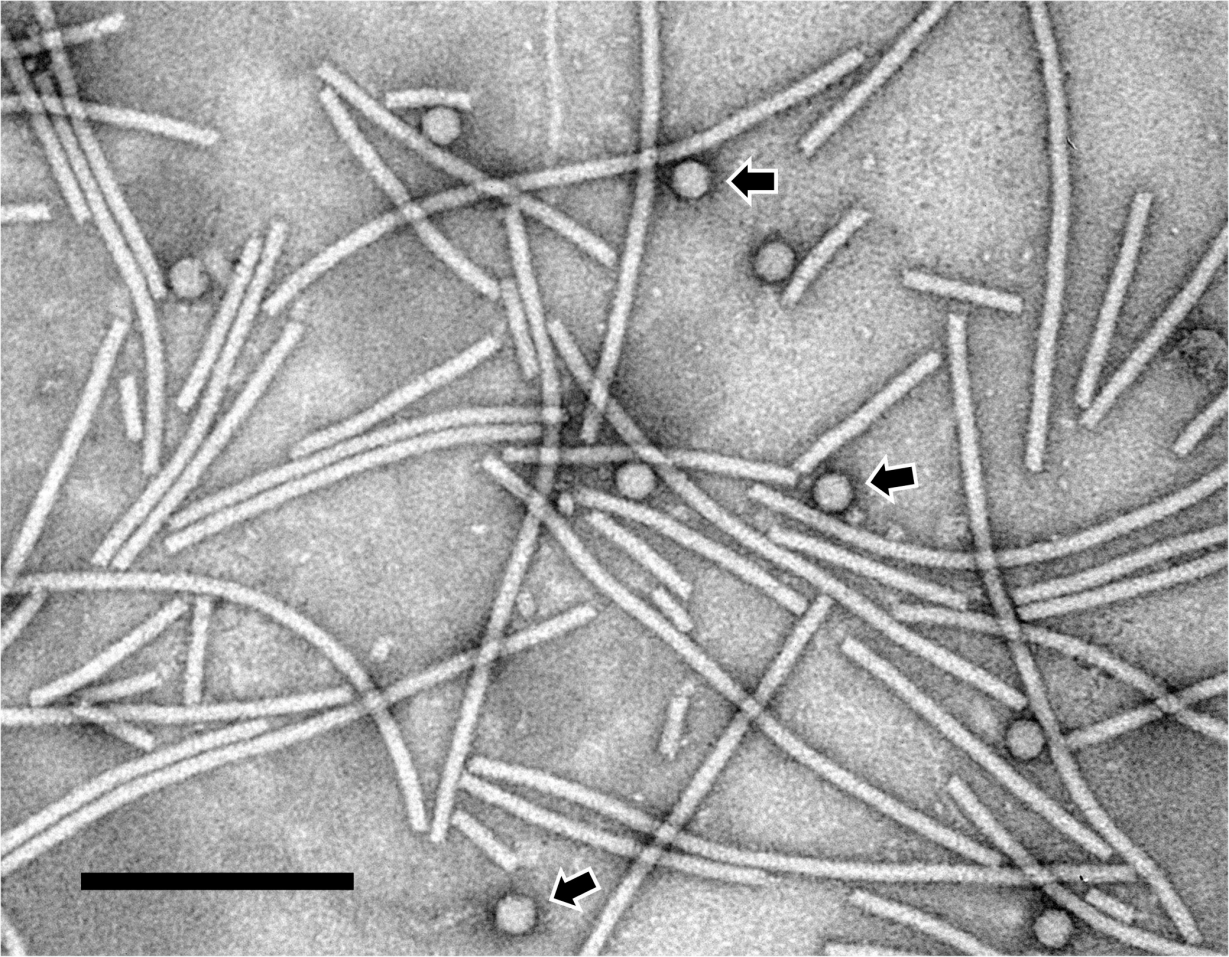
Transmission electron micrograph of partially purified Miscanthus yellow fleck virus virus-like particles. The coat protein of Miscanthus yellow fleck virus was transiently expressed in *Nicotiana benthamiana* using a potato virus X-based vector. The flexuous, rod-shaped particles correspond to *Potato virus X*, and arrows indicate the presence of icosahedral Miscanthus yellow fleck virus virus-like particles. Black bar corresponds to 200 nanometers.

To functionally validate the CP sequence, it was heterologously expressed in the model plant *Nicotiana benthamiana* using a PVX-based vector. VLPs were purified from leaf tissue and analyzed by transmission electron microscopy. Long, flexuous rods were visible and were interpreted to correspond to PVX, as expected given the use of a PVX-based vector. Icosahedral -shaped VLPs were also visible in the sample, and these were attributed to the expression of the CP of MYFV (Fig 4). *Luteoviridae* viruses are known to produce icosahedral virus particles of 24–30 nanometers (nm) in diameter [13], and the viral particles observed for MYFV fit within this description with the VLPs having an average diameter of 28 nm.

## Conclusion

In this paper, we identify and describe a new polerovirus that is associated with yellow flecks on leaves of *Miscanthus sinensis*. This virus has all the genomic characteristics of poleroviruses and clearly groups in a phylogenetic tree within poleroviruses. All identified proteins have less than 90% identity to those of known poleroviruses, and, therefore, it qualifies as a new virus in the *Luteoviridae* family per the species demarcation criteria (≥10% difference in amino acid sequence of any gene) [14]. We subsequently refer to this virus as Miscanthus yellow fleck virus (MYFV).

MYFV is closely related to the previously characterized poleroviruses ScYLV and WLYaV. ScYLV is a virus of economic importance on sugarcane; since *Miscanthus sinensis* is being used in sugarcane breeding, there is a possibility that hybrid miscanes could serve as a common host for these two viruses. Furthermore, identification of MYFV allows for the development of diagnostic tools that are important for successful plant regulatory and quarantine procedures. Interestingly, diagnostic primers developed for ScYLV CP sequence also worked to amplify MYFV CP sequence. Future research should focus on identifying the vector, host range, and genetic diversity for this new polerovirus.
